# Supplementary material for: Single-cell spatial mapping reveals alteration of tissue microenvironment during early colorectal cancer
Source: bioRxiv. 2025 Aug 27:2024.11.20.622725. Originally published 2024 Nov 21. Preprint. [Version 2] doi: 10.1101/2024.11.20.622725 (PMC11601668; doi:10.1101/2024.11.20.622725)
Supplement: Supplement 1 [file NIHPP2024.11.20.622725v2-supplement-1.pdf]

bioRxiv preprint doi: https://doi.org/10.1101/2024.11.20.622725; this version posted August 27, 2025. The copyright holder for this preprint (which was not certified by peer review) is the author/funder, who has granted bioRxiv a license to display the preprint in perpetuity. It is made available under aCC-BY 4.0 International license.

Supplemental information

Document S1. Figures S1 – S4, Tables S1 – S3

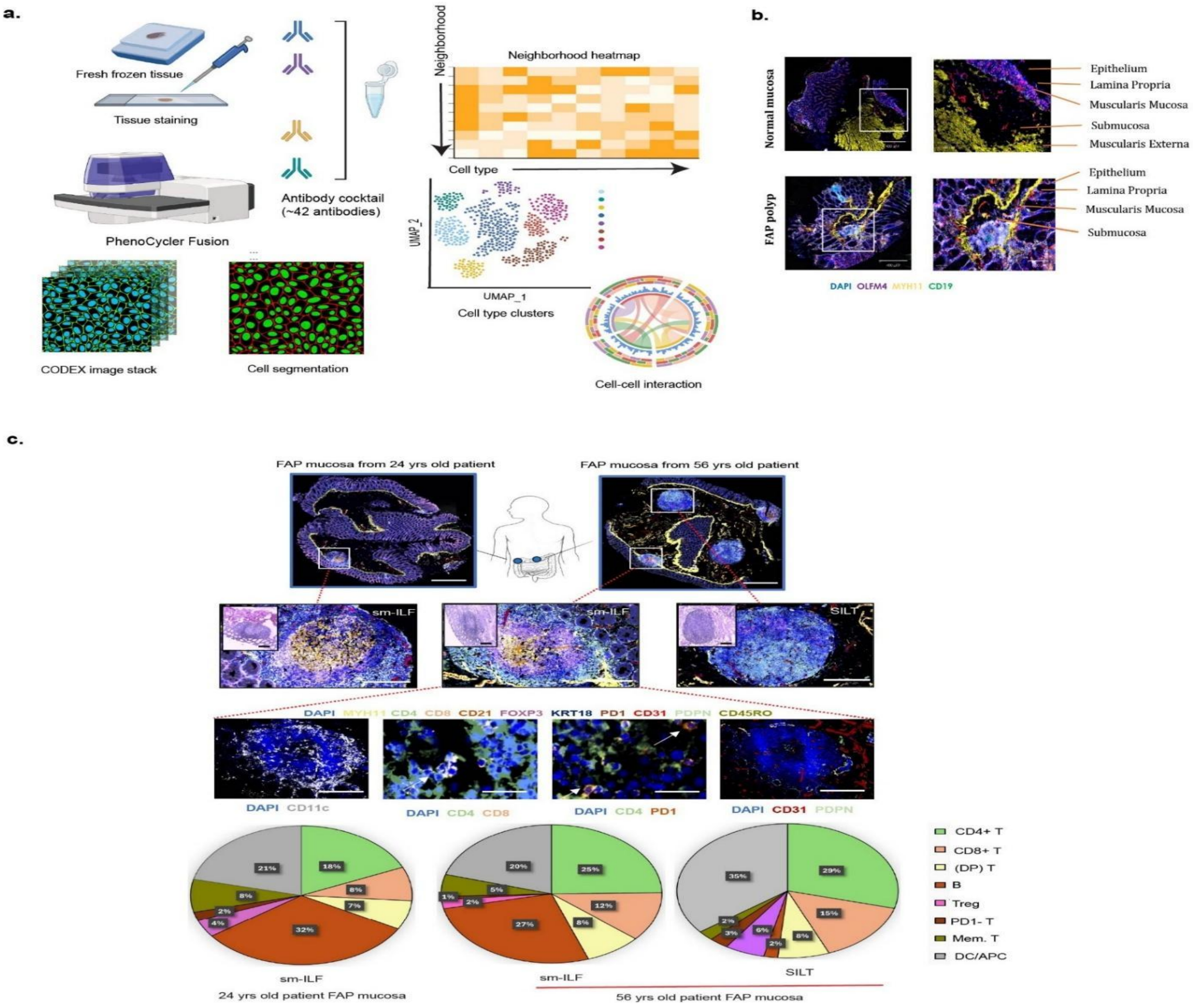

Figure S1: Detecting overall colon tissue morphology and lymphoid follicles using standard CODEX workflow

bioRxiv preprint doi: https://doi.org/10.1101/2024.11.20.622725; this version posted August 27, 2025. The copyright holder for this preprint (which was not certified by peer review) is the author/funder, who has granted bioRxiv a license to display the preprint in perpetuity. It is made available under aCC-BY 4.0 International license.

(a) Schematic representation of CODEX workflow (see text for details). (b) Representative CODEX images showing overall tissue morphology from normal mucosa (upper panel) and FAP mucosa (lower panel), scale bar: 200 μm (c) CODEX images of FAP mucosa samples from 24 year old and 56 year old FAP patients, showing the presence of submucosal intestinal lymphoid follicles (sm-ILF) and solitary intestinal lymphoid tissue (SILT), scale bar: 200 μm. CODEX detects the presence of dendritic cells (DC), CD4+ T cells, CD8+ T cells, Double-positive (DP)- T cells and microcapillaries within the sm-ILF. Lymphatic endothelial cells are present in the periphery, scale bar: 50 μm. Pie charts representing the percentages of various immune cell types within these lymphoid structures have been provided.

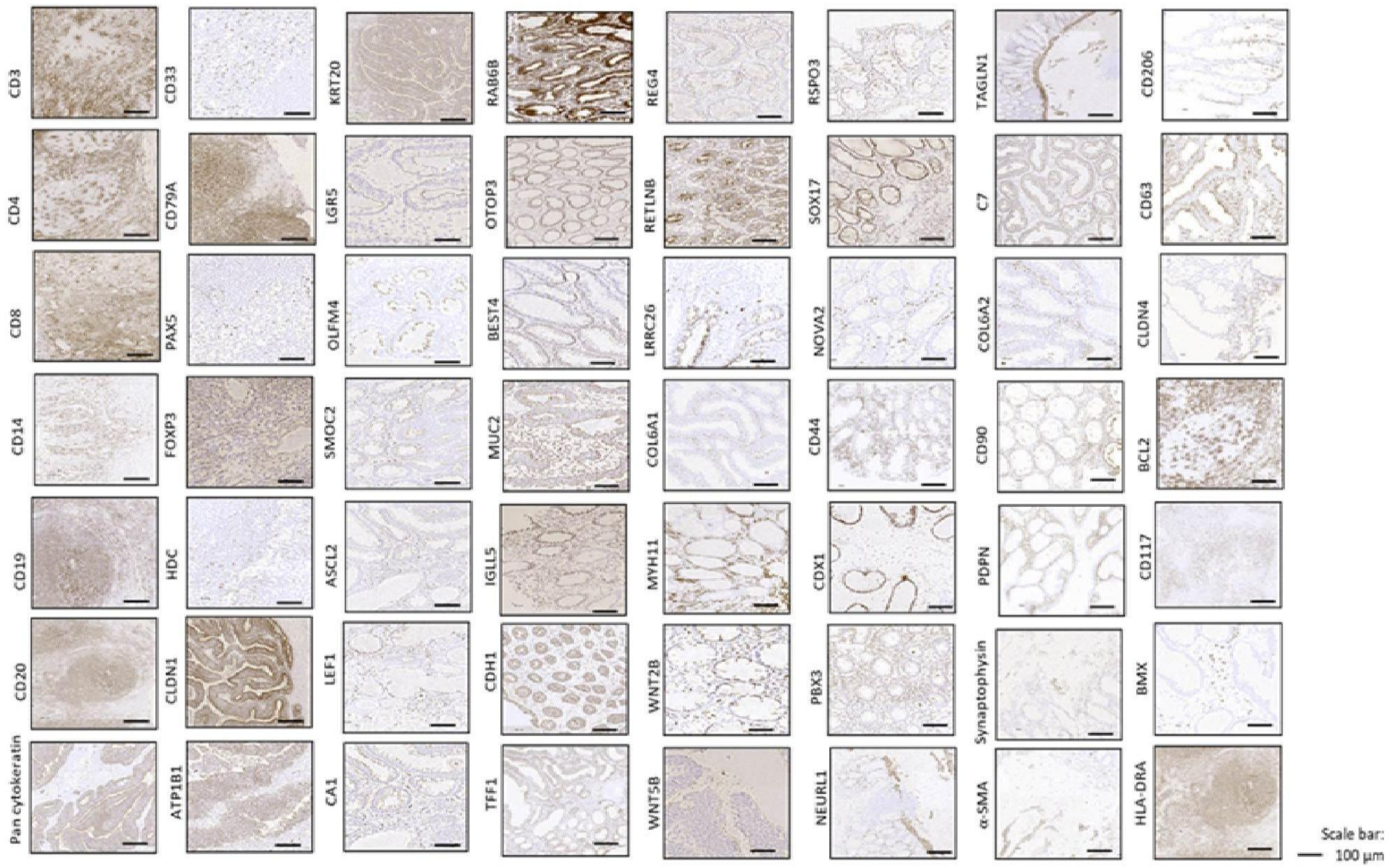

Figure S2: Antibody validation

Individual antibody validation performed on positive control tissues and colon tissues before including in the CODEX marker panel for performing CODEX experiments.

bioRxiv preprint doi: https://doi.org/10.1101/2024.11.20.622725; this version posted August 27, 2025. The copyright holder for this preprint (which was not certified by peer review) is the author/funder, who has granted bioRxiv a license to display the preprint in perpetuity. It is made available under aCC-BY 4.0 International license.

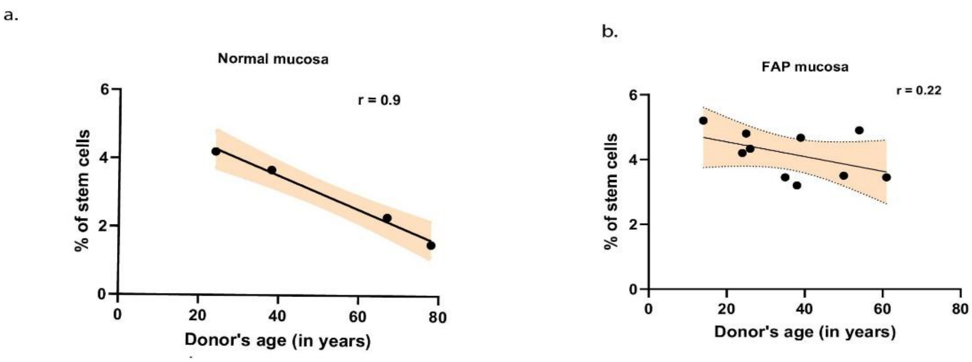

Figure S3: Correlation of stem cells found in polyp samples and donors' age

Correlation of intestinal stem cell population with donor's age from (**a**) normal mucosa: Strong correlation has been observed (r = 0.9) indicating stem cells decrease with increase in age within normal mucosa samples (**b**) no such correlation has been observed (r = 0.22) between age and stem cell population derived from FAP mucosa samples, indicating stem cells proliferate irrespective of the age of the patient.

bioRxiv preprint doi: https://doi.org/10.1101/2024.11.20.622725; this version posted August 27, 2025. The copyright holder for this preprint (which was not certified by peer review) is the author/funder, who has granted bioRxiv a license to display the preprint in perpetuity. It is made available under aCC-BY 4.0 International license.

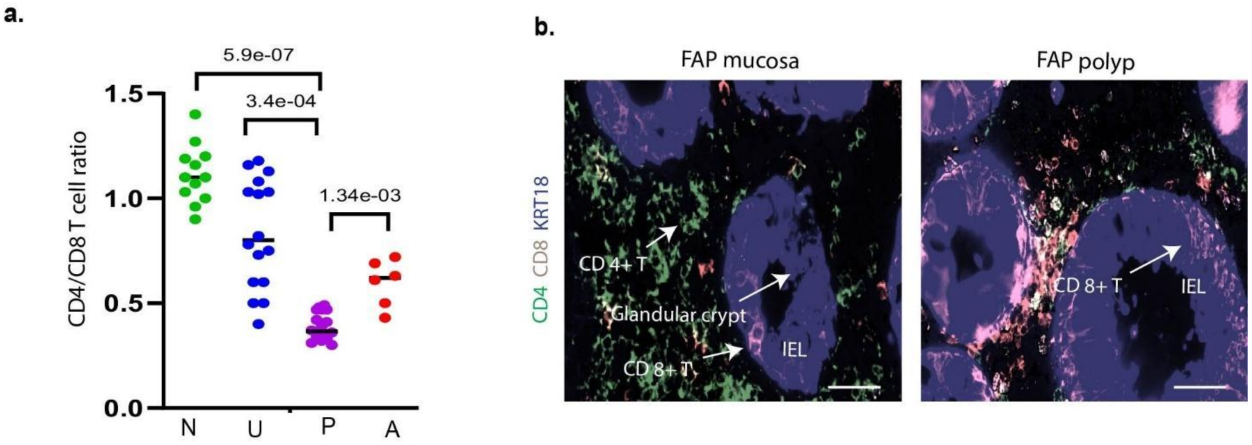

Figure S4: CD4+T and CD8+ T cell type composition changes across FAP disease continuum

(**a**) Scattered dot plots representing the CD4/BD8 T cell ratio across normal mucosa (N), FAP mucosa (U), FAP polyp (P) and, FAP adenocarcinoma/CRC (A). A two-tailed statistical t test was performed and adjusted P value has been indicated, (**b**) CODEX imaging showing CD8+ T cell tend to localize near the epithelial glandular crypts as intraepithelial lymphocytes (IEL). This is mostly observed in FAP polyps compared to FAP mucosa. Scale bar: 10µm

Donor study ID	Disease status	Current/ Age at collection	Sex	Race/Ethnicity	Cancer Status	Sample collected	Underlying health condition
A001	FAP (clinical dx)	50 / 45	Male	Hispanic/Latino	Sigmoid colon cancer	Colectomy	Asthma
A002	FAP (APC+)	24 / 20	Female	White	No cancer	Colectomy	Unknown
A014	FAP (clinical dx, negative genetic test)	25 / 22	Female	white	No cancer	Colectomy	Childhood asthma
A015	FAP (APC+)	38 / 36	Female	Hispanic/Latino	No cancer	Colectomy	Unknown
A018	FAP (APC+)	56 / 53	Male	Hispanic/Latino	No cancer	Colonoscopy	Hypertension
A035	FAP (clinical dx, negative genetic test)	61 / 58	Male	White	Desmoid Tumor	Colonoscopy	Hypertension
A040	FAP	14 / 12	Male	Hispanic/Latino	No cancer	Colectomy	Unknown
A051	FAP	26 / 25	Male	Hispanic/Latino	No cancer	Colectomy	Diabetes mellitus
A055	FAP	39 / 35	Female	White	No cancer	Colectomy	Asthma

bioRxiv preprint doi: https://doi.org/10.1101/2024.11.20.622725; this version posted August 27, 2025. The copyright holder for this preprint (which was not certified by peer review) is the author/funder, who has granted bioRxiv a license to display the preprint in perpetuity. It is made available under aCC-BY 4.0 International license.

A057	FAP (clinical dx, negative genetic test)	54 / 52	Male	Hispanic/Latino	No cancer	Colectomy	Unknown
A069	Adenocarcinoma	65 / 67	Male	White	Colorectal cancer	Colectomy	Unknown
G	FAP	35 / 32	Male	Hispanic/Latino	Colorectal cancer	Colectomy	Unknown
CRC_15564	Adenocarcinoma, medullary type	Unknown / 66	Female	White	Colorectal cancer	Tumor	Unknown
CRC_8396	Adenocarcinoma, medullary type	34 / 31	Male	Hispanic/Latino	Colorectal cancer	Tumor	Unknown
CRC_23135	Adenocarcinoma, medullary type	Unknown / 45	Female	White	Colorectal cancer	Tumor	Unknown
CRC_19779	Adenocarcinoma, medullary type	57 / 54	Male	Hispanic/Latino	Colorectal cancer	Tumor	Unknown
B001	Normal mucosa	67	Female	White	Healthy colon	Autopsy	Diabetes, hypertension
B004	Normal mucosa	78	Male	Black	Healthy colon	Autopsy	Diabetes, hypertension
B005	Normal mucosa	24	Female	White	Healthy colon	Autopsy	None
B006	Normal mucosa	38	Male	White	Healthy colon	Autopsy	None

Table S1: Donor metadata.

Twelve FAP and eight non-FAP donors consented for this study. See text for details.

bioRxiv preprint doi: https://doi.org/10.1101/2024.11.20.622725; this version posted August 27, 2025. The copyright holder for this preprint (which was not certified by peer review) is the author/funder, who has granted bioRxiv a license to display the preprint in perpetuity. It is made available under aCC-BY 4.0 International license.

General Paper ID	HTAN/HuBMAP Biospecimen ID	Donor	FAP	Pathology	Location	Storage
A001-C-002	HTA10_01_002	A001	Y	FAP polyp (P)	Descending	FF
A001-C-007	HTA10 01 007	A001	Y	FAP Adenocarcinoma (A)	Descending	OCT
A001-C-023	HTA10_01_023	A001	Y	FAP mucosa (U)	Descending	FF
A001-C-043	HTA10 01 043	A001	Y	FAP mucosa (U)	Descending	FF
A001-C-104	HTA10_01_104	A001	Y	FAP polyp (P)	Tranverse	FF
A002-C-016	HTA10 02 016	A002	Y	FAP polyp (P)	Descending	FF
A002-C-021	HTA10_02_021	A002	Y	FAP polyp (P)	Descending	FF
A002-C-024	HTA10 02 024	A002	Y	FAP mucosa (U)	Descending	FF
A002-C-106	HTA10_02_106	A002	Y	FAP polyp (P)	Tranverse	FF
A002-C-212	HTA10_02_212	A002	Y	FAP mucosa (U)	Ascending	FF
A014-C-002	HTA10_03_002	A014	Y	FAP polyp (P)	Rectum	FF
A014-C-114	HTA10_03_114	A014	Y	FAP mucosa (U)	Tranverse	FF
A015-C-005	HTA10_04_005	A015	Y	FAP polyp (P)	Rectum	FF
A015-C-010	HTA10_04_010	A015	Y	FAP mucosa (U)	Descending	FF
A015-C-202	HTA10_04_202	A015	Y	FAP polyp (P)	Ascending	FF
A015-C-204	HTA10_04_204	A015	Y	FAP polyp (P)	Ascending	FF
A015-C-206	HTA10_04_206	A015	Y	FAP mucosa (U)	Ascending	FF
A015-C-207	HTA10_04_207	A015	Y	FAP mucosa (U)	Ascending	FF
A015-C-208	HTA10_04_208	A015	Y	FAP mucosa (U)	Ascending	FF
A018-E-014	HTA10_18_014	A018	Y	FAP polyp (P)	Ascending	FF
A018-E-021	HTA10_18_021	A018	Y	FAP polyp (P)	Transverse	FF
A035-E-011	HTA10_18_011	A035	Y	FAP mucosa (U)	Ascending	FF
A035-E-045	HTA10_35_045	A035	Y	FAP polyp (P)	Descending	FF
A040-C-005	HTA10 40 005	A040	Y	FAP mucosa (U)	Descending	FF
A040-C-006	HTA10_40_006	A040	Y	FAP polyp (P)	Descending	FF
A051-C-058	HTA10 51 058	A051	Y	FAP polyp (P)	Descending	FF
A051-C-059	HTA10_51_059	A051	Y	FAP mucosa (U)	Descending	FF
A055-C-212	HTA10 55 212	A055	Y	FAP mucosa (U)	Ascending	FF
A055-C-213	HTA10_55_213	A055	Y	FAP polyp (P)	Ascending	FF
A057-C-112	HTA10 57 112	A057	Y	FAP polyp (P)	Tranverse	FF
A057-C-113	HTA10_57_113	A057	Y	FAP mucosa (U)	Tranverse	FF
A057-C-115	HTA10 57 115	A057	Y	FAP mucosa (U)	Tranverse	FF
A057-C-116	HTA10_57_116	A057	Y	FAP polyp (P)	Tranverse	FF
A069-C-052	HTA10 69 052	A069	Y	FAP Adenocarcinoma (A)	Descending	FF
G_Desc_3	HTA10_6_003	G	Y	FAP mucosa (U)	Descending	FF
G024	HTA10 6 024	G	Y	FAP polyp (P)	Descending	FF
CRC_8396	HTA10_10_109	CRC1	N	Sporadic CRC (A)	Transverse	FF
CRC 15564	HTA10 11 112	CRC2	N	Sporadic CRC (A)	Ascending	FF
CRC_19779	HTA10_12_115	CRC3	N	Sporadic CRC (A)	Descending	FF
CRC 23135	HTA10 13 118	CRC4	N	Sporadic CRC (A)	Descending	FF
B001-A-001	HBM945.PZHJ.983	B001	N	Normal mucosa (N)	Mid-jejunum	FF
B001-A-101	HBM683.LPTG.838	B001	N	Normal mucosa (N)	Tranverse	FF
B001-A-201	HBM369.JRRC.377	B001	N	Normal mucosa (N)	Ascending	FF
B001-A-301	HBM773.SCDC.559	B001	N	Normal mucosa (N)	Sigmoid	FF
B001-A-401	HBM682.TMBK.865	B001	N	Normal mucosa (N)	Tranverse	FF
B004-A-004	HBM294.KLBX.557	B004	N	Normal mucosa (N)	Descending	FF
B004-A-008	HBM777.LKTF.999	B004	N	Normal mucosa (N)	Descending	FF
B004-A-404	HBM998.TTXD.949	B004	N	Normal mucosa (N)	Mid-jejunum	FF
B004-A-408	HBM896.LDWJ.896	B004	N	Normal mucosa (N)	Jejunum	FF
B004-A-504	HBM965.TTFH.425	B004	N	Normal mucosa (N)	Duodenum	FF
B005-A-002	HBM662.ZXJN.782	B005	N	Normal mucosa (N)	Descending	FF
B006-A-101	HBM633.PRRH.456	B006	N	Normal mucosa (N)	Tranverse	FF

Table S2: Sample metadata used in this study

Fifty-two samples including 12 normal mucosa, 16 FAP mucosa, 18 FAP polyp, 2 FAP adenocarcinoma and, 4 CRCs were selected for CODEX imaging and analyses. These samples are color coded (see text for details).

bioRxiv preprint doi: https://doi.org/10.1101/2024.11.20.622725; this version posted August 27, 2025. The copyright holder for this preprint (which was not certified by peer review) is the author/funder, who has granted bioRxiv a license to display the preprint in perpetuity. It is made available under aCC-BY 4.0 International license.

Table S3. Details of CODEX oligo-barcoded antibodies used in this study

Antibody name	rr ID	Uniprot accesior	Dilution	Akoya barcod	Flouresent tag	Akoya Catalog	Custom conjugatio	Validation
Anti-CD3 antibody	AB 2744378	P09396	1/100	BX015	Alexa Fluor™ 647	4550103	N	Y
Anti-CD4 antibody	AB 2728838	P01730	1/100	BX021	Alexa Fluor™ 647	4550105	N	Y
Anti-CD8 antibody	AB 2744463	P01732	1/100	BX026	Atto 550	4250012	N	Y
Anti-CD14 antibody	AB 2737723	P08571	1/50	BX040	Alexa Fluor™ 488	5150009	Y	Y
Anti-CD19 antibody	AB 2864513	P25918	1/100	BX003	Alexa Fluor™ 647	4550099	N	Y
Anti-CD20 antibody	AB 2737014	P11836	1/100	BX007	Alexa Fluor™ 488	4150018	N	Y
Anti-Pan cytokeratin antibody	AB_2133034	Q04695	1/25	BX019	Alexa Fluor™ 488	4150020	N	Y
Anti-CD33 antibody	AB_2738045	P20138	1/100	BX034	Alexa Fluor™ 488	5150007	Y	Y
Anti-PAX5 antibody	AB_2159686	Q02548	1/200	BX042	Alexa Fluor™ 647	5550015	Y	Y
Anti-FOXP3 antibody	AB 2738006	Q9BZS1	1/100	BX031	Alexa Fluor™ 647	4550071	N	Y
Anti-HDC antibody	AB 591860	P19113	1/50	BX047	Atto 550	5250009	Y	Y
Anti-CLDN1 antibody	AB 387598	O88551	1/50	BX042	Alexa Fluor™ 647	5550015	Y	Y
Anti-ATP1B1 antibody	AB 11205684	P05026	1/25	BX049	Alexa Fluor™ 488	5150012	Y	Y
Anti-KRT20 antibody	AB 2920709	P35900	1/50	BX013	Alexa Fluor™ 488	5450017	Y	Y
Anti-LGR5 antibody	AB 2737881	O75473	1/100	BX005	Atto 550	5450024	Y	Y
Anti-OLFM4 antibody	AB 1294582	Q6UX06	1/100	BX017	Atto 550	5250001	Y	Y
Anti-SMOC2 antibody	AB_2286471	Q9H3U7	1/100	BX020	Atto 550	5250002	Y	Y
Anti-ASCL2 antibody	AB_841910	Q99929	1/100	BX006	Alexa Fluor™ 647	5550018	Y	Y
Anti-LEF1 antibody	AB_1546369	Q9UJU2	1/200	BX014	Atto 550	5450025	Y	Y
Anti-CA1 antibody	AB 11205000	P00915	1/200	BX010	Alexa Fluor™ 488	5450016	Y	Y
Anti-RAB6B antibody	AB 2689796	Q9NRW1	1/100	BX033	Alexa Fluor™ 647	5550013	Y	Y
Anti-OTOP3 antibody	AB 1854837	Q7RTS5	1/100	BX030	Alexa Fluor™ 647	5550012	Y	Y
Anti-BEST4 antibody	AB 2683759	Q8NFU0	1/50	BX013	Alexa Fluor™ 488	5450017	Y	Y
Anti-MUC2 antibody	AB 10898088	Q02817	1/100	BX022	Alexa Fluor™ 488	5150003	Y	Y
Anti-IGLL5 antibody	AB 2511443	B9A064	1/100	BX045	Alexa Fluor™ 647	5550016	Y	Y
Anti-CDH1 antibody	AB 10563376	Q6R8F2	1/50	BX014	Atto 550	4250021	N	Y
Anti-TFF1 antibody	AB_2202851	P04155	1/100	BX041	Atto 550	5250008	Y	Y
Anti-REG4 antibody	AB_2805293	Q9BYZ8	1/100	BX017	Atto 550	5250001	Y	Y
Anti-RETLNB antibody	AB_2178024	Q9BQ08	1/50	BX015	Alexa Fluor™ 647	5550008	Y	Y
Anti-LRRC26 antibody	AB 10913866	Q2I0M4	1/100	BX002	Atto 550	5450023	Y	Y
Anti-COL6A1 antibody	AB 2045451	P12109	1/200	BX014	Atto 550	5450025	Y	Y
Anti-MYH11 antibody	AB 2736180	P35749	1/200	BX029	Atto 550	5250005	Y	Y
Anti-WNT2B antibody	AB 856692	Q93097	1/200	BX025	Atto 550	5250015	Y	Y
Anti-RSPO3 antibody	AB 11205697	Q9BXY4	1/100	BX040	Atto 550	5250017	Y	Y
Anti-SOX17 antibody	AB 10893402	Q9H6I2	1/100	BX031	Alexa Fluor™ 488	5150006	Y	Y
Anti-NOVA2 antibody	AB 2282703	Q9UNW9	1/200	BX030	Alexa Fluor™ 647	5550012	Y	Y
Anti-CD44 antibody	AB_2244424	P16070	1/100	BX005	Atto 550	4450041	N	Y
Anti-PBX3 antibody	AB_2160479	P40424	1/50	BX027	Alexa Fluor™ 647	5550011	Y	Y
Anti-NEURL1 antibody	AB_10751198	O76050	1/100	BX037	Alexa Fluor™ 488	5150008	Y	Y
Anti-TAGLN1 antibody	AB 2617350	Q5BKM2	1/200	BX026	Atto 550	5250004	Y	Y
Anti-C7 antibody	AB 10896185	P10643	1/100	BX049	Atto 550	5250019	Y	Y
Anti-COL6A2 antibody	AB 1294123	P12110	1/200	BX024	Alexa Fluor™ 647	5550010	Y	Y
Anti-CD90 antibody	AB 852827	P04216	1/100	BX022	Alexa Fluor™ 488	4150021	N	Y
Anti-PDPN antibody	AB 11205535	Q86YL7	1/50	BX121	Atto 550	4250094	N	Y
Anti-Synaptophysin antibody	AB 2336016	Q16563	1/100	BX016	Alexa Fluor™ 488	5150001	Y	Y
Anti-alpha SMA antibody	AB 11220108	P62736	1/100	BX006	Alexa Fluor™ 647	5550017	Y	Y
Anti-CD206 antibody	AB_10896526	P22897	1/100	BX042	Alexa Fluor™ 647	5550015	Y	Y
Anti-CD63 antibody	AB_394205	P08962	1/50	BX045	Alexa Fluor™ 647	5550016	Y	Y
Anti-CLDN4 antibody	AB_2768947	O14493	1/100	BX007	Alexa Fluor™ 488	5450015	Y	Y
Anti-BCL2 antibody	AB 786068	P10415	1/200	BX030	Alexa Fluor™ 647	5550012	Y	Y
Anti-BMX antibody	AB 2290762	P51813	1/100	BX033	Alexa Fluor™ 647	5550013	Y	Y
Anti-HLA DR antibody	AB 10565677	P01903	1/50	BX033	Alexa Fluor™ 647	4550118	N	Y
Anti-beta catenin antibody	AB 2861317	P35222	1/100	BX096	Atto 550	4250091	N	Y
Anti-CEA antibody	AB 2229224	P13688	1/50	BX001	Alexa Fluor™ 488	5450013	Y	Y
Anti-MKi67 antibody	AB 2770367	P46013	1/100	BX047	Atto 550	4250019	N	Y
Anti-VCAN antibody	AB 2904000	P13611	1/100	BX050	Alexa Fluor™ 647	5550003	Y	Y
Anti-CD45 antibody	AB_2174555	P08575	1/50	BX001	Alexa Fluor™ 488	4150003	N	Y
Anti-CD68 antibody	AB_2750607	P34810	1/200	BX015	Alexa Fluor™ 647	4550113	N	Y
Anti-CD69 antibody	AB_2738120	Q07108	1/100	BX041	Atto 550	4250022	N	Y

While some antibodies were purchased directly from Akoya vendor, most of them were conjugated with oligo barcodes in-house and validated as per the manufacturer's protocol (see Methods for details).
